# Comparison of the Width of Peritumoral Surgical Margin in Open and Robotic Partial Nephrectomy: A Propensity Score Matched Analysis

**DOI:** 10.1371/journal.pone.0158027

**Published:** 2016-06-23

**Authors:** Jong Jin Oh, Jung Keun Lee, Kwangmo Kim, Seok-Soo Byun, Sang Eun Lee, Sung Kyu Hong

**Affiliations:** Department of Urology, Seoul National University Bundang Hospital, Seongnam, Korea; Eberhard Karls University, GERMANY

## Abstract

**Background:**

To compare the surgical margin status after open partial nephrectomy (OPN) and robotic partial nephrectomy (RPN) performed in patients with T1a renal cell carcinoma (RCC).

**Methods:**

This was a propensity score-matched study including 702 patients with cT1a RCC treated with partial nephrectomy (PN) between May 2003 and July 2015. Perioperative parameters, including surgical margin width after PN, were compared between two surgical methods. After determining propensity score for tumor size and location, the width of peritumoral surgical margin was investigated. Multivariate logistic analysis to predict peritumoral surgical margin less than 1mm was analyzed.

**Results:**

The mean width of peritumoral surgical margin was 2.61 ± 2.15 mm in OPN group (n = 385), significantly wider than the 2.29 ± 2.00 mm of RPN group (n = 317) (p = 0.042). The multivariate analysis showed surgical methods was significant factors to narrow surgical margin less than 1mm (p = 0.031). After propensity score matching, the surgical margin width was significantly longer in OPN (2.67 ± 2.14 mm) group than RPN (2.25 ± 2.03 mm) group (p = 0.016). A positive resection margin occurred in 7 (1.8%) patients in the OPN group and 4 (1.3%) in the RPN group. During the median follow-up of 48.3 months, two patients who underwent OPN had tumor bed recurrence.

**Conclusions:**

RPN may result in a narrower peritumoral surgical margin than OPN. Further investigation on the potential impact of such a phenomenon should be performed in a larger-scale study.

## Introduction

Currently, partial nephrectomy (PN) has become the typical method for treating single renal tumors.[1, 2] Several studies have shown that for treating renal cell carcinoma (RCC) tumors <4 cm in their greatest dimension, nephron sparing sugery (NSS) offers equally effective local control and a similar disease-specific survival rate compared to a radical nephrectomy (RN) [1, 3]. Moreover, a PN could spare normal functional renal tissue of the ipsilateral kidney and provide definite tumor excision, which provided advantage in aspect of renal functional preservation [4, 5].

As with any oncologic surgery, the surrogate for determining a complete tumor resection during a PN is a negative surgical margin (NSM). Although PN prevents the loss of function in the renal functional mass, it carries the risk of incomplete tumor excision. A positive surgical margin (PSM) is a worrisome issue during a PN [6]. Early studies raised concerns that in cases of tumors with high malignant potential, PSMs are closely associated with a higher recurrence rate [7]. Although several subsequent studies showed that PSMs have negligible or no impact on tumor recurrence and metastasis [8], the concern about local recurrence resulting from inadequate tumor excision remains. Nevertheless, in recent years, there have been several reports on reducing the width of safety margins, such as a tumor enucleation technique that consists of excising the tumor using blunt dissection without a visible rim of normal parenchyma [9].

With the introduction of the robotic surgical approach, which provided three-dimensional, high-definition magnified vision, robotic partial nephrectomy (RPN) is increasingly being performed PN for the management of small renal masses [10, 11]. With the advantage of articulated robotic instruments and wider vision, it is possible to excise closer to the tumor and preserve more renal parenchymal tissue during RPN compared with an open partial nephrectomy (OPN) [12]. Meanwhile, in that sense, it can be hypothesized that RPN, compared with OPN, may result in narrowing of peritumoral surgical margin, possibly contributing to an increased risk of surgical margin positivity. Thus, we compared the pathologic outcomes, including width of peritumoral surgical margin and margin positivity, after OPN and RPN in T1a RCC using propensity score matching analysis.

## Materials and Methods

### Ethics Statement

The study was approved by our institutional review board, Seoul National University Bundang Hospital Institutional review board and follows the rules stated in the Declaration of Helsinki. All participants gave written informed consent and were reimbursed for their participation.

### Study Population

After obtaining approval from the institutional review board, we reviewed the records of RCC patients who underwent an OPN or RPN from May 2003 to July 2015. We included patients with a clinical T1a renal tumor measuring less than 4 cm and pathologic renal cell carcinoma. We excluded patients with a single kidney, bilateral renal masses, von Hippel-Lindau syndrome or an OPN under hypothermia and cold ischemia. We also excluded patients who had a metastasis at the time of the PN or a benign tumor after the PN and excluded the initial 30 RPN cases. In total, 702 patients were enrolled in this analysis.

### Data Collection

The 702 patients were divided into two groups based on the surgical method. Of these patients, 385 patients (54.8%) underwent an OPN and 317 patients (45.2%) underwent an RPN. Prior to the PN, baseline data were collected, including age, sex, body mass index (BMI), tumor laterality (right or left), tumor size, tumor location and preoperative renal function. The tumor location was defined as exophytic, endophytic, mesophytic, or hilar [13].

### Follow Up Strategy after PN

For patients who underwent a PN, the kidney CT, blood test including renal functional data and chest x-ray were performed in postoperative 3 months according to guideline [14]. We re-checked these examinations in postoperative 12 months after surgery and after then, we performed annually.

### Histopathologic Evaluation

Surgical PN specimens were processed using standard pathologic techniques and were reviewed by a single genitourinary pathologist. All tumors were graded according to the Fuhrman nuclear grading system [15]. Tumors were measured by their maximal diameter and were staged according to the 2010 American Joint Committee on Cancer classification system.

### Margin Evaluation

A PSM was defined as malignant cells being present at the inked parenchymal surgical margin of resection on the final pathology assessment. Normal parenchymal tissue around the resection margin, regardless of thickness, was considered to be an NSM. In each case, the maximal and minimal distances from the cut edge of the renal parenchyma to the tumor were measured. The width of the peritumoral surgical margin was defined as the minimal length. When no renal parenchyma was present outside the pseudocapsule, the surgical margin width was recorded as zero.

### Statistical Analyses

Clinical and pathological covariates were evaluated using a Chi-squared test for categorical variables. A Mann–Whitney test was used for continuous variables. After propensity score matching using preoperative parameters of the two groups, the peritumoral surgical margin widths were compared. Multivariate logistic regression analysis to predict a narrow surgical margin less than 1mm after PN was conducted including surgical methods.

## Results

### Baseline Characteristics of the OPN and RPN Patients

The median age of the 702 patients enrolled in the study was 53 years. The patients who underwent an OPN were older (54.88 vs 52.13 years, p = 0.004), had larger tumors (23.05 vs 21.68 mm, p = 0.029) and had lower preoperative renal function (glomerular filtration rate (GFR) 77.51 vs 91.35 ml/min) than the RPN group (Table 1). Renal tumors resected using RPN were more often exophytic. In the RPN group, 203 renal tumors (64.0%) were exophytic. In the OPN group, 189 renal tumors (49.1%) were exophytic.

**Table 1:** Demographic characteristics of study patients according to surgical methods in clinical T1a renal tumor.

Characteristics	OPN (n = 385)	RPN (n = 317)	p-value
Age (y) ± SD	54.88 ± 13.08	52.13 ± 12.24	0.004
Sex (%)			0.392
Male	268 (69.6)	230 (72.6)	
Female	117 (30.4)	87 (27.4)	
Body mass index (kg/m^2^) ± SD	24.55 ± 3.03	24.73 ± 3.34	0.449
Mean ASA score ± SD	1.64 ± 0.60	1.58 ± 1.53	0.205
Tumor laterality (%)			0.974
Right	195 (50.6)	156 (49.2)	
Left	190 (49.4)	161 (50.8)	
Tumor size (mm) ± SD(IQR)	23.05 ± 8.36 (16.00–30.00)	21.68 ± 8.19 (15.00–27.50)	0.029
Tumor location (%)			0.003
Exophytic	189 (49.1)	203 (64.0)	
Mesophytic	65 (16.9)	41 (12.9)	
Endophytic	120 (31.2)	68 (21.5)	
Hilar	11 (2.8)	5 (1.6)	
Preopertive Cr (mg/dl) ± SD	0.98 ± 0.27	0.88 ± 0.21	< 0.001
Preopertive MDRD-GFR (ml/min) ± SD	77.51 ± 18.58	91.35 ± 56.61	< 0.001

OPN; open partial nephrectomy, RPN; robotic partial nephrectomy, SD; standard deviation, ASA; American Society of Anesthesiologists, Cr; creatinine, GFR; glomerular filtration rate

### Pathological, Perioperative Outcomes and Surgical Margin Width

As shown in Table 2, the operative times were similar between the two groups. However, the warm ischemic time (WIT) was shorter in the OPN group than in the RPN group (17.30 vs 20.59 min, p < 0.001). The estimated blood loss and postoperative complications were more favorable in the RPN group. The final pathologic stage and grade were similar between the two groups. Ten OPN patients had a pathologic score of T3a. Two RPN patients had pathologic score of T3a (p = 0.089). Most of the resected renal tumors were clear cell RCC. Their Fuhrman grades were 2 or 3.

**Table 2:** Perioperative and pathological outcomes after open partial nephrectomy and robotic partial nephrectomy among clinical T1a tumor.

Characteristics	OPN (n = 385)	RPN (n = 317)	P value
Perioperative outcomes			
Operative time (min)	140.15 ± 46.83	138.83 ± 72.44	0.772
Warm ischemic time (min)	17.30 ± 7.37	20.59 ± 7.61	< 0.001
EBL (mL)	214.26 ± 202.66	167.16 ± 236.63	0.006
Transfusion (%)	12 (3.1)	5 (1.6)	0.187
Intraoperative complications (%)	14 (3.6)	11 (3.5)	
Postoperative complications (%)	40 (10.4)	15 (4.7)	0.008
Clavein-Dindo classification ≥ 3 (%)	27 (7.0)	7 (2.2)	
Pathologic outcomes			
Pathologic T stage (%)			0.089
T1a	375 (97.4)	315 (99.4)	
T3a	10 (2.6)	2 (0.6)	
Furhman’s grade (%)			0.421
1	7 (1.8)	3 (0.9)	
2	178 (46.2)	132 (41.6)	
3	149 (38.7)	135 (42.6)	
4	12 (3.1)	7 (2.2)	
Resection margin positive (%)	7 (1.8)	4 (1.7)	0.555
Safety margin (mm) ± SD	2.61 ± 2.15	2.29 ± 2.00	0.042
Clear cell type RCC (%)	290 (75.3)	241 (76.0)	0.210

OPN; open partial nephrectomy, RPN; robotic partial nephrectomy, EBL; estimated blood loss, RCC; renal cell carcinoma

PSM occurred in 7 (1.8%) patients in the OPN group and 4 patients (1.3%) in the RPN group (p = 0.555). The surgical margin width was 2.61 ± 2.15 mm in the OPN group, which was significantly wider than the surgical margin of 2.29 ± 2.00 mm in the RPN group.

### Multivariate Logistic Regression Analysis to Predict Narrow Surgical Margin

As shown in Table 3, multivariate logistic regression analysis to predict narrow peritumoral surgical margin of less than 1mm showed BMI, tumor size, tumor location and surgical methods. Patients who had a larger tumor size (odds ratio (OR) = 0.943, p < 0.001) and an exophytic renal mass (OR = 0.341, p < 0.001) had significantly negative associations with having a narrow surgical margin of less than 1mm. OPN compared with RPN had a negative association with narrower surgical margin in this analysis (OR = 0.666, p = 0.031).

**Table 3:** Multivariate analysis on the surgical margin less than 1mm among patients who underwent partial nephrectomy.

	OR	95% CI	*p* value
Body mass index	1.056	1.001–1.114	0.047
Tumor size	0.943	0.922–0.964	< 0.001
Tumor location (exophytic vs others)	0.341	0.238–0.488	< 0.001
Tumor side (Right vs left)	1.075	0.764–1.511	0.678
Ischemic time	1.018	0.992–1.044	0.182
Surgical methods (open vs robot)	0.666	0.461–0.964	0.031

### Surgical Margin Width after Propensity Score Matched Analysis

We re-analyzed the data using propensity score matching to reduce the potential bias. The results are shown in Table 4. In a matched cohort analysis, all the preoperative parameters were similar between the two cohorts. Although the tumor size and tumor location were similar, the peritumoral surgical margin width was significantly larger in the OPN group (2.67 ± 2.14 mm) than in the RPN group (2.25 ± 2.03 mm, p = 0.016).

**Table 4:** Comparison of perioperative data according to surgical methods after propensity score matching.

Characteristics	OPN (n = 299)	RPN (n = 299)	p-value
Age (y) ± SD	53.25 ± 12.87	52.89 ± 12.02	0.723
Sex (%)			0.324
Male	205 (68.6)	216 (72.2)	
Female	94 (31.4)	83 (27.8)	
Body mass index (kg/m^2^) ± SD	24.58 ± 2.98	24.78 ± 3.32	0.440
Tumor laterality (%)			0.964
Right	147 (49.2)	148 (49.5)	
Left	152 (50.8)	151 (50.5)	
Tumor size (mm) ± SD(IQR)	22.36 ± 8.19 (15.00–28.00)	21.99 ± 8.20 (15.00–28.00)	0.580
Tumor location (%)			0.554
Exophytic	172 (57.5)	185 (61.9)	
Mesophytic	47 (15.7)	41 (13.7)	
Endophytic	71 (23.7)	68 (22.7)	
Hilar	9 (3.1)	5 (1.7)	
Operative time (min)	140.89 ± 46.21	137.45 ± 59.06	0.428
Warm ischemic time (min)	17.01 ± 7.69	20.76 ± 7.66	<0.001
Resection margin positive (%)	5 (1.67)	4 (1.33)	0.500
Peritumoral surgical margin (mm) ± SD	2.67 ± 2.14	2.25 ± 2.03	0.016

OPN; open partial nephrectomy, RPN; robotic partial nephrectomy, SD; standard deviation

### Surgical Margin Width and Cancer Recurrence

**Fig 1:**
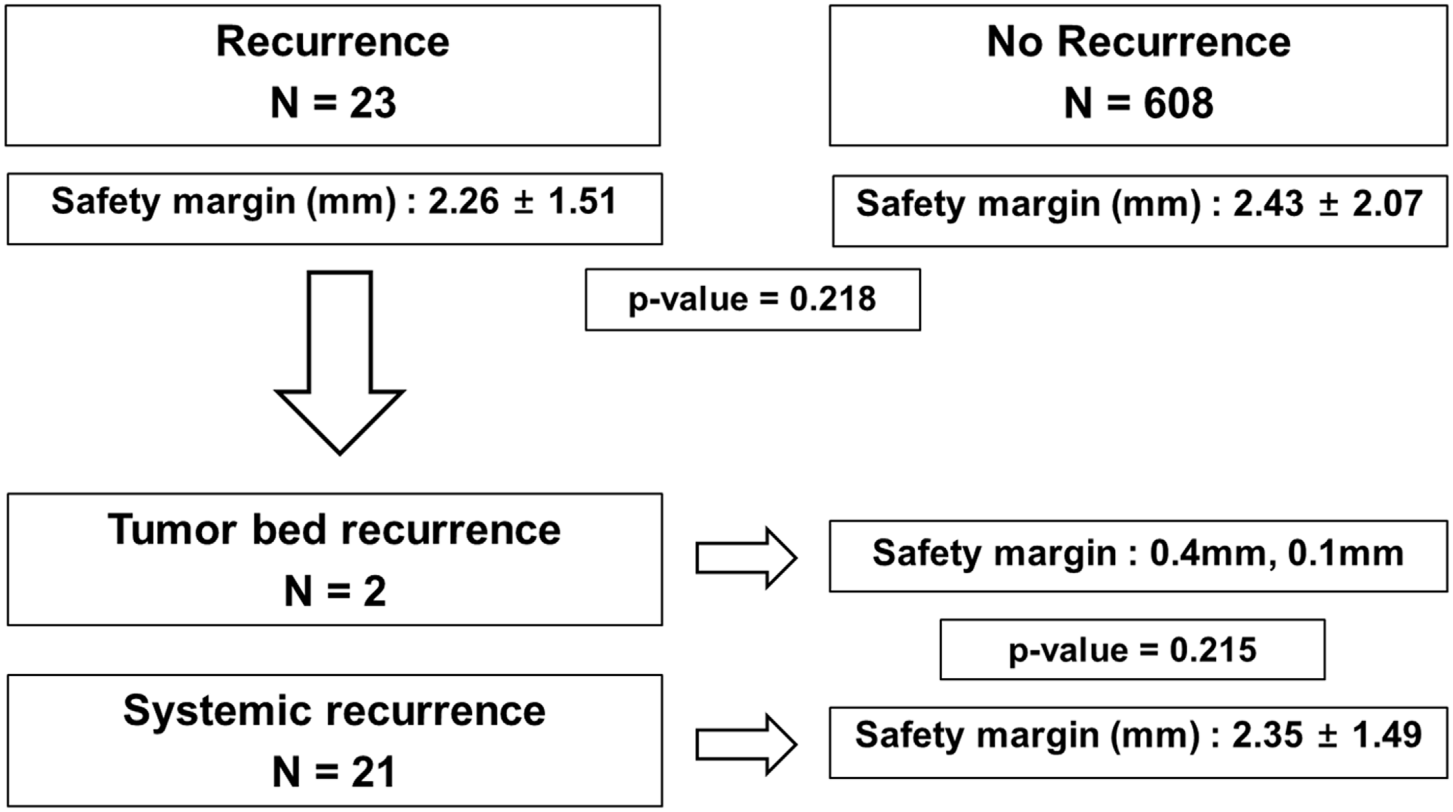
Comparison of the peritumoral surgical margin width according to cancer recurrence after a partial nephrectomy in 702 patients with clinical T1a renal cell carcinomas.

During the median follow-up period of 48.3 months, tumor recurrence occurred in 23 patients (3.3%). The mean surgical margin at the time of the PN in patients with recurrence was 2.26 ± 1.51 mm. In patients without recurrence, the margin was 2.43 ± 2.07 mm (p = 0.218) (Fig 1). Of the 23 patients with cancer recurrence, only two patients had tumor bed recurrence in the OPN cohort. The other 21 patients had systemic recurrence, which may have been metastasis. The surgical margin was negative in the two patients who had surgical bed recurrence. The peritumoral surgical margin widths in the two patients with surgical bed recurrence were 0.4 mm and 0.1 mm.

## Discussions

In the current study, we observed that, for T1a RCC, the width of the surgical margin was narrower after RPN than after OPN. After propensity score matching, differences in surgical margin width between OPN and RPN remained statistically significant. Meanwhile, the two groups demonstrated no significant difference in the rate of PSM. Although several studies showed that peritumoral surgical margin width was not significant in oncologic outcomes, we focused on surgical margin width during PN, especially in the robotic platform era that provided much more magnified view of surgical field compared with OPN.

To prevent the risk of local cancer recurrence, excision of an adequate visible margin of the normal-appearing parenchyma around the tumor is considered to be the standard surgical technique of PN [2]. However, the surgical margin width that should be removed with the tumor during PN remains controversial. An optimal margin will guarantee complete tumor removal and minimize local recurrence rates. An over-resected margin could increase surgical difficulty and compromise residual renal function, especially in solitary kidney. It could also increase morbidity from surgical complications [16]. The resection of tumor with 1-cm safety margin of normal-appearing parenchyma around the tumor has been considered the standard surgical technique for PN for many years [17]. However, this concept has been challenged by a series of recent studies. In a prospective study of Stage pT1a RCC treated with a radical nephrectomy, positive cancer lesions beyond the pseudocapsule in 19.5% of patients, with an average distance from the primary tumor of 0.5 mm [18]. They concluded that when PN is performed, 5-mm margin is enough to prevent local recurrence. In another retrospective study of 69 patients who had undergone PN, Castilla *et al*. [19] found that a histologic tumor-free resection margin was sufficient to achieve complete local RCC control, and resection margin width did not correlate with long-term disease progression. More recently, in a retrospective study with 115 patients, Li *et al*. [16] showed that a mini-margin of less than 5 mm was safe and there was only 1 local recurrence. In our analysis, the mean width of the surgical margin in the total cohort was only 2.45 mm, relatively narrow compared with findings from other analyses. Meanwhile, there were only two local recurrences in our analysis. Such a finding would be supportive of the assumption that a mini-margin of less than 3 mm is relatively safe in PN with a clinical T1a RCC.

Not many have performed comparative analysis of peritumoral surgical margins of OPN and RPN. Some published series on partial nephrectomy have shown that the surgical margin width after RPN was relatively narrower than in the OPN series [12, 20]. Potentially, such phenomenon may pose an oncologic risk as the number of RPN performed worldwide is rapidly increasing. Looking at the literature, several published series have shown that the PSM rates of an OPN and an RPN are not significantly different [19, 21, 22]. However, there is a limitation of the generalizability of such findings because most of such data originated from high-volume institutions [23, 24]. One population-based cohort study showed that after a PN, the PSM was approximately 10% [25]. A recent large-scale study using a national cancer database with 11,587 patients with clinical T1a RCC showed a PSM prevalence of 4.9% following an OPN and 8.7% following an RPN (p < 0.001) [24]. They also concluded that a higher PN surgical volume appeared to reduce the occurrence of PSM in the top quartile of PN surgical volume (> 55 cases per year) centers and had significantly low incidences of PSM compared to centers in the lower three quartiles. The observed higher rate of PSM from RPN may be a product of the initial learning curve. Thompson *et al*. [26] recently reported the outcomes from a single surgeon’s experience of robotic surgery and found that PSM rates were initially high but decreased as experience increased.

Similarly the effect of an initial learning curve could contribute to narrower surgical margin width during RPN. To reduce the bias from the learning curve, we excluded the initial 30 cases in the PRN series because 30 cases had been mentioned as an adequate number to overcome the initial learning curve in RPN [27–29]. Thus more plausible explanation for the finding from our study would be the magnified view of surgeon during RPN. As surgeons obtain much more magnified view during RPN compared with OPN, they may be prone to have inaccurate assessment of peritumoral margin during RPN. As robotic platform provides a stable, clear and magnified view around tumor, such effect may well result in surgeons inching closer to tumor than ever before. Although to a lesser degree, loss of tactile feedback during RPN may also be a factor behind our finding. It should be reminded that, in RPN, excised tumor mass cannot be examined immediately as can be done in OPN.

Our study is the first propensity score matched study comparing surgical margin width between an OPN and an RPN. We found that the surgical margin width was significantly narrower in the RPN group than the OPN group. Meanwhile, we observed that PSM and the recurrence rates were not different between the two groups. It can be suggested that our marginal status result may have been different with a larger cohort because a relatively higher PSM rate for RPN has recently been reported from a population-based study. Chen *et al*. [30] showed that the rates of cancer lesions beyond the pseudocapsule rate were 39% in T1b RCC and 25% in T1a RCC. The extra-pseudocapsular cancer was located within 3.0 mm of the primary tumor. Therefore, they concluded that the surgical margin width should be greater than 3.0 mm, despite the presence of an NSM. Micrometastasis has been mentioned as another reason for maintaining a safe surgical margin width. For example, studies in other solid cancers, such as hepatic cellular carcinoma, showed that a wide margin resection was safer than a narrow margin width, due to the possibility of micrometastasis around the tumor [31]. Although no significant association was noted between the width of surgical margin and the margin positivity, we believe that our findings provide alarming information for surgeons performing RPN.

This study had some limitations. First, it was a retrospective cohort study. Therefore, selection bias could be present. However, we used a propensity score matched analysis to reduce bias. Secondly, only few patients experienced cancer recurrence. Therefore, we did not analyze oncological outcomes using peritumoral surgical margin width. To overcome these limitations, we should build population-based registry at a national level which importance was reviewed in article by Pearson *et al*. [32]. The large scaled, national leveled, population-based database was mandatory for the future kidney cancer study.

## Conclusions

After adjusting for tumor size and location by propensity score matching in clinical T1a renal tumors, we observed that the width of surgical margin was narrower in RPNs than in OPNs. Although the rate of PSM was not found to be significantly different between OPN and RPN group in our series, further evaluation on the potential impact of a narrower surgical margin in RPN should be performed in a larger-scale study.
